# Genome Mining Revealed a High Biosynthetic Potential for Antifungal *Streptomyces* sp. S-2 Isolated from Black Soot

**DOI:** 10.3390/ijms21072558

**Published:** 2020-04-07

**Authors:** Piotr Siupka, Artur Piński, Dagmara Babicka, Zofia Piotrowska-Seget

**Affiliations:** Institute of Biology, Biotechnology and Environmental Protection, Faculty of Natural Sciences, University of Silesia in Katowice, 40-032 Katowice, Poland; apinski@us.edu.pl (A.P.); dagmarabbb@gmail.com (D.B.); zofia.piotrowska-seget@us.edu.pl (Z.P.-S.)

**Keywords:** antifungal activity, biosynthetic gene clusters, black soot, coal-related environments, genome mining, *Streptomyces*

## Abstract

The increasing resistance of fungal pathogens has heightened the necessity of searching for new organisms and compounds to combat their spread. *Streptomyces* are bacteria that are well-known for the production of many antibiotics. To find novel antibiotic agents, researchers have turned to previously neglected and extreme environments. Here, we isolated a new strain, *Streptomyces* sp. S-2, for the first time, from black soot after hard coal combustion (collected from an in-use household chimney). We examined its antifungal properties against plant pathogens and against fungi that potentially pose threat to human health (*Fusarium avenaceum*, *Aspergillus niger* and the environmental isolates *Trichoderma* citrinoviridae Cin-9, *Nigrospora oryzae* sp. roseF7, and *Curvularia coatesieae* sp. junF9). Furthermore, we obtained the genome sequence of S-2 and examined its potential for secondary metabolites production using anti-SMASH software. The S-2 strain shows activity against all of the tested fungi. Genome mining elucidated a vast number of biosynthetic gene clusters (55), which distinguish this strain from closely related strains. The majority of the predicted clusters were assigned to non-ribosomal peptide synthetases or type 1 polyketide synthetases, groups known to produce compounds with antimicrobial activity. A high number of the gene clusters showed no, or low similarity to those in the database, raising the possibility that S-2 could be a producer of novel antibiotics. Future studies on *Streptomyces* sp. S-2 will elucidate its full biotechnological potential.

## 1. Introduction

*Streptomyces*, a genus of Actinobacteria, includes over 800 species described so far [1]. These bacteria are Gram-positive, have a large genome (8–10 Mbp) with high GC content, have a complex morphology, and are widespread in soil, where they play important roles in the degradation of organic matter and nutrient circulation (for a review, see [2]).

The members of this genus are known producers of a wide variety of secondary metabolites that are important in medicine and industry [2,3,4]. *Streptomyces* are a major source for antibiotics used by humans, as they produce over two-thirds of the clinically useful antibiotics that are of natural origin [1,5,6,7,8,9]. Moreover, it has been estimated that *Streptomyces* potentially produce approximately 100,000 antimicrobial metabolites, of which only a small portion have been identified [5,7]. Furthermore, they produce many other ecologically and biotechnologically important molecules such as pigments, biologically active compounds, and exoenzymes [3,10,11]. In nature, microbial secondary metabolites are an important part of ecological and physiological interactions with the environment and within microbial communities. They allow *Streptomyces* to outcompete other microorganisms as well as overcome environmental stressors [12,13]—roles that are crucial especially in extreme environments [13,14,15]. Advancements in sequencing and genome mining methods have shown that *Streptomyces* have greater than previously predicted potential to produce secondary metabolites, as a large number of biosynthetic gene clusters (BGCs) have been revealed [5,16,17,18,19]. Often, more than 15% of the *Streptomyces* genome is dedicated to secondary metabolism [20]. Most BGCs remain uncharacterized, and the peptides that they encode may synthesize new antibiotics [17]. The expression of BGCs is tightly regulated, and many remain in a dormant stage [2,4,18,19,21,22]. Recently, the activation of BGCs from *Streptomyces sclerotialus* NRLP ISP-5269 using genetic engineering has led to the discovery of scleric acid, a new compound that is active against *Mycobacterium tuberculosis* [23].

Extensive antibiotic resistance among bacteria and fungi is one of the major challenges in medicine and agriculture. The need to constantly battle new pathogens has led to searches of previously neglected and extreme environments for new strains that show antibiotic activity [1,13,24,25]. For instance, several *Streptomyces* strains with antibiotic activity have been isolated from a marine sponge [26]. A thermotolerant strain showing antimicrobial potential, *Streptomyces* sp. TM32, has been recently reported [17]. Furthermore, the novel alkaliphilic strain, *Streptomyces* sp. McG1, has been shown to inhibit the growth of ESKAPE pathogens (*Enterococcus feacium*, *Staphylococcus aureus*, *Klebsiella pneumoniae*, *Acinetobacter baumani*, *Pseudomonas aeruginosa*, and *Enterobacter* spp.) [27]. *Streptomyces* strains with antimicrobial activity have been found in insect microbiomes [25] as well as in Antarctica [13]. Many members of *Streptomyces* have antifungal properties [2,3,25,28,29,30,31,32,33]. *Streptomyces varsoviensis* produces hygrobafilomycin, an antifungal macrolide with a unique molecular structure [34]. A new species, *Streptomyces fodineus*, has shown antifungal activity against a broad range of filamentous fungi. The strain was first isolated from acidic soil collected from an abandoned coal mine area in Korea [1]. This points to mines and environments related to fossils as sources of new strains with antibiotic activity.

A number of filamentous fungi are common pathogens of crops and other plants important for agriculture and other industries [35,36,37]. Members of the genus *Fusarium* are examples of such pathogens. *Fusarium graminearum* infects wheat, barley, and maize, causing losses for food and brewing industries [38]. Another species, *Fusarium oxysporum*, is a pathogen with numerous potential hosts [36]. It causes Fusarium wilt of banana disease, one of the most destructive plant diseases of the modern era [35]. *Fusarium avenaceum*, another pathogen with numerous hosts, can cause infections in crops, peas, or other plants [39,40,41]. *Nigrospora* is another genus whose species are common pathogens of plants [42,43,44,45]. Fighting fungal infections is thus of high importance. The use of synthetic fungicides can place additional pressure on the environment, and their accumulation can cause unpredicted toxic effects. Furthermore, their efficiency and the possibility of using them is influenced by seasonal weather conditions. Therefore, researchers are trying to find natural biocontrol agents and natural antifungal products [2,28,32,37]. The biological control of Fusarium wilt of banana has been investigated using both bacteria and other fungi (for a review, see Reference [35]). Actinobacteria have also been studied for their ability to protect plants from fungal infections [2,28,35,37,46,47]. One example is *S. griseorubiginouse*, which has been shown to be effective against Fusarium wilt of banana [48]. However, this research was limited to in vitro conditions and no field experiments were conducted [35]. *S. lydicus* WYEC 108 is part of a bio fungicide that has been approved by the Environmental Protection Agency (EPA) for use in the United States (EPA registration number 73314-1) [49]. *S. violaceusniger* YCED9 produces three antifungal compounds that are effective against plant pathogens [50]. Nevertheless, a continuous search for new antibiotic-producing strains and new compounds with biological activity against plant pathogens is important within the scope of constant development of resistance to existing agents. Genome mining can be used to reveal gene clusters, leading to the synthesis of a large plethora of new compounds and advancing the discovery of new antibiotics. Moreover, newly discovered strains and compounds might be beneficial to the health of humans as well as to livestock and household animals. A number of filamentous fungi pose a health hazard as they release mycotoxins that can cause severe diseases and death [32,51,52]. This group includes members of *Aspergillus* and *Trichoderma,* [32,51,53,54] and the genus *Fusarium*, which can cause systemic infections [55]. New, natural fungicides can potentially be of use for the reduction of exposure to mycotoxins, or could lead to new fungicides that can help treat humans or animals.

The aim of this study was to examine the antifungal potential of a new strain, *Streptomyces* sp. S-2, which was isolated from black soot after coal combustion (collected from a household chimney). The influence of this strain on filamentous fungi, including plant pathogens, was investigated. The genome of the new strain was sequenced and mined to elucidate the strain biosynthetic potential of secondary metabolites. *Streptomyces* sp. S-2 exhibited strong antifungal activity against all tested fungi. Genome mining revealed a large number of biosynthetic gene clusters suggesting that the strain has the potential to produce novel compounds with biological activity.

## 2. Results

### 2.1. The Isolation of the Streptomyces sp. S-2 Strain From Black Soot

**Figure 1:**
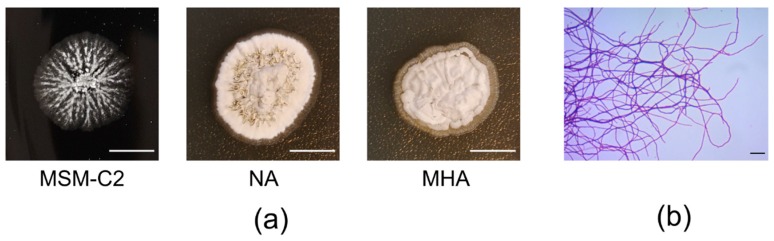
Morphology of *Streptomyces* sp. S-2 colonies. (**a**) Growth in solid cultures on 1% hard coal dust (MSM-C2), nutrient agar (NA), and Mueller—Hinton agar (MHA) plates. For the initial culture *Streptomyces* sp. S-2 was streak-inoculated on agar plates. The plates were incubated at 30 °C for 3 days, after which pictures of the colonies were taken. The scale bar represents 5 mm (**b**) Bacterial mycelia stained using the Gram method. After 3 days, the colonies on the MSM-C2 agar plates were scraped using inoculation loop and suspended in 0.85% NaCl, dried, and stained using the Gram method, followed by observation with a light microscope under immersion, using a Plan-ACHROMAT 100× 1.25NA objective (Carl Zeiss, Germany). Pictures were recorded using an AxioCam ERc 5s (Carl Zeiss, Germany). The scale bar represents 10 μm. All pictures were processed using Fiji software.

Here, 1 g of the black soot sample was spread onto a minimal salt medium (MSM) agar plate supplemented with 1% hard coal dust (MSM-C2). After two weeks, a thin, white circlet appeared around the piece of soot. This was collected and spread onto a fresh MSM-C2 agar plate. After 3 days, colonies with a compact structure and creamy-white protrusions appeared, growing within the media. Single colonies were spread onto nutrient agar (NA) and Mueller—Hinton agar (MHA) plates and were used for Gram staining (Figure 1). On each medium, the colonies exhibited a typical *Streptomyces* morphology that was compact and white on the MSM-C2 and slightly brown on the NA and MHA. There were creamy-white protrusions on the MSM-C2 and creamy-white, wrinkled plaque on the NA and MHA plates (Figure 1A). Gram staining showed Gram-positive bacteria with a mycelial-like morphology (Figure 1B). Additionally, after 3 days, the colonies emitted an earthy/forest-like odor, which is another distinct feature of *Streptomyces*. The strain also stained culture media releasing a dark brown pigment. This occurred even when it was kept in pure culture. The strain was used for further characterization.

### 2.2. Phenotypic Fingerprint of Streptomyces sp. S-2

To characterize *Streptomyces* sp. S-2, 8-day-old cell cultures were scraped from the MSM-C2 plates and inoculated on GENIII microplate assay plates. The growth of the bacteria was measured 48 h post-inoculation (Table 1 and Table S1). The strain is able to utilize 42 out of the 71 compounds in the assay as a source of carbon and energy. These compounds included basic sugars, disaccharides dextrins, and polyols (glycerol and D-mannitol). The strain is also capable of utilizing several amino acids and a number of organic acids, including polycarboxylic acids. It has a proteolytic feature (the utilization of gelatin) and the ability to degrade polysorbates (Tween 40). The *Streptomyces* sp. S-2 tolerates slight-to-moderate halophilic conditions (growth at an NaCl concentration of 1% to 8% with a reverse correlation to salt concentration) and slightly acidic conditions (growth at pH 6 but not pH 5). It is susceptible to all of the tested antibiotics except for nalidixic acid and Aztreonam. The strain is able to grow in the presence of denaturing agents (guanidine HCl) and desiccants (lithium chloride), features that could be connected to adaptation to extreme and arid environments, such as the black soot from the household chimney.

### 2.3. Antifungal Activity of Streptomyces sp. S-2

The antifungal activity of *Streptomyces* sp. S-2 was tested against two fungi from culture collections—*Aspergillus niger* and *Fusarium avenaceum*—and three fungi from the environment, including two pathogens that were isolated from a garden rose and a juniper. Fungal strains isolated from the environment were selected for the study based on their different morphologies in order to obtain diverse genera. The selected fungi were identified by sequencing the internal transcribe spacer (ITS) region and searching the National Center for Biotechnology Information NCBI database using a Blast tool. All sequences were deposited in the NCBI database. The fungi used in the study were from a cinder sample: *Trichoderma citrinoviridae* Cin-9 (MN756678), from a garden rose: *Nigrospora oryzae* roseF7 (MN756677), and from a juniper: *Curvularia coatesieae* junF9 (MN756676). 

**Figure 2:**
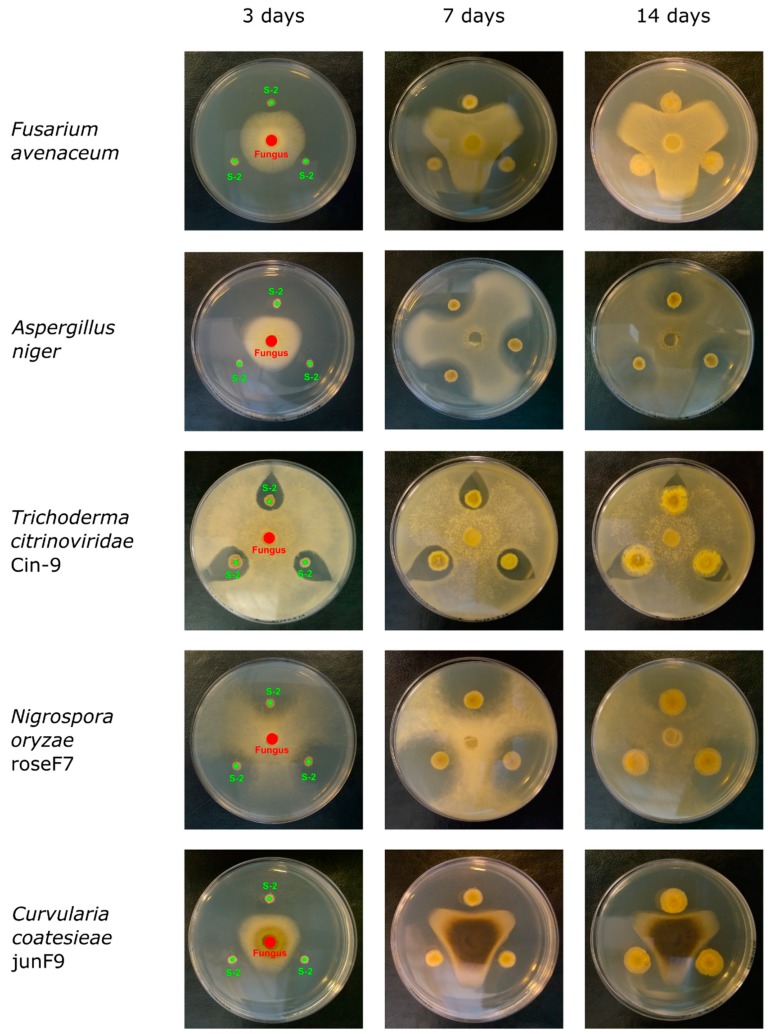
The inhibition of fungal growth by *Streptomyces* sp. S-2. Representative pictures of the S-2 strain and each fungus co-culture showing the inhibition of fungal mycelia growth taken at 3rd, 5th, and 14th day of the cultures. For each co-culture, MHA plates were used except for the co-culture with *Trichoderma citroviridae* Cin-9, which was performed on NA plates. The S-2 strain was spot-inoculated at the 20 mm distance from the center of plate, followed by placing a puck of agar with fungal mycelium at the center of the plate. Plates were incubated at 25 °C, except for the co-culture with *T. citrinoviridae* Cin-9, which was incubated at 30 °C. Co-cultures were investigated daily and pictures of the co-cultures were taken. For each co-culture, at least 14 plates were analyzed.

**Figure 3:**
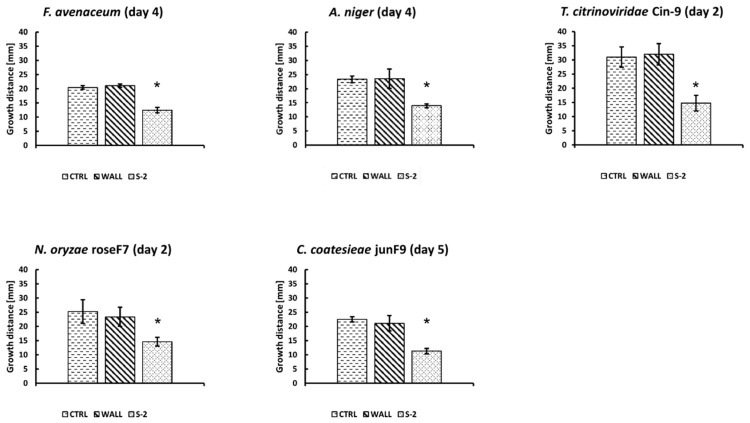
Inhibition of fungal growth by *Streptomyces* sp. S-2. Charts comparing the growth of fungal mycelia towards the plate border (WALL) or toward the S-2 strain colonies (S-2) on co-culture plates and fungal mycelia growth toward the border of the plates on control plates (CTRL). For each co-culture, MHA plates were used except for the co-culture with *T. citroviridae* Cin-9 which was performed on NA plates. The S-2 strain was spot-inoculated at a distance 20 mm from the center of the plate, followed by the placement of a puck of agar with fungal mycelia at the center of the plate. Plates were incubated at 25 °C, except for the co-culture with *T. citrinoviridae* Cin-9 which was incubated at 30 °C. For each co-culture, at least 14 plates were analyzed. Three control plates for fungal growth control for each experimental set were also analyzed. A growth comparison for each fungus on the day when fungal mycelia grew ≥ 20 mm (the distance at which the S-2 strain was inoculated) is indicated in brackets above the charts. Statistically significant inhibitions of growth are represented by an asterisk (*) (one-way ANOVA followed by Tukey’s post hoc test, *p* ≤ 0.05).

To test the antifungal activity, the S-2 strain was simultaneously co-cultured with fungi on MHA or NA plates. The plates were cultured at 25 °C, a temperature optimal for fungi growth, except for co-cultures with *T. citrinoviridae* Cin-9, which were cultured at 30 °C. Fungi growth was investigated every day for 14 days. Representative pictures of the co-cultures are shown in Figure 2. Antifungal activity was measured by comparing the inhibition of mycelial growth towards the center of the S-2 colonies to growth towards the border of the plates. The S-2 strain exhibited antifungal activity against all of the tested fungi, but at different levels of inhibition (Figure 3; data not shown). The effect was visible early in the co-cultures with the fast-growing fungi (*T. citrinoviridae* Cin-9 and *N. oryzae* roseF7). The highest antifungal activity was observed against *N. oryzae* roseF7, where, over time, a visual inspection of plates indicated that compactness of the mycelium decreased (data not shown), suggesting lysis of the fungi. A strong inhibitory effect was also observed for *C. coatesieae* junF9. A moderate effect was observed for *F. avenaceum*. For *T. citrinoviridae* Cin-9 and *A. niger*, even though there was a high level of inhibition observed at the beginning of the co-culture, during the course of the experiment, both fungi were able to nearly completely cover the plates with mycelia (Figure 2). Importantly, when the fungal mycelia reached the S-2 colonies, further growth could not be measured even though the fungi could have been viable. Thus, this was a situation in which measuring growth distances did not reflect actual effects. Therefore, to better compare the effects of S-2 on the tested fungi, the relative growth inhibition was measured on the day when the mycelia that were growing towards the plate border reached a distance of S-2 inoculation spots, 20 mm from the plate center (Figure 3). For *N. oryzae* roseF7 and *T. citrinoviridae* Cin-9 this was on day 2 post-inoculation, for *A. niger* and *F. avenaceum* this occurred on day 4 post-inoculation, and for *C. coatesieae* junF9 this was on day 5 post-inoculation. At the point when the mycelia reached that distance, the strongest inhibitory effect was observed against *T. citrinoviridae* Cin-9 (53.84% ± 7.17%; *n* = 15), followed by *C. coatesieae* junF9 (45.97% ± 4.67%; *n* = 18), then *F. avenaceum* (40.81% ± 5.97%; *n* = 15), and *A. niger* (39.86% ± 7.86%; *n* = 14), and finally *N. oryzae* roseF7 (35.54% ± 14.17%; *n* = 16) (Figure 3).

To further investigate antifungal activity and overcome the issues with the co-cultures that were mentioned above, *Streptomyces* sp. S-2 was inoculated in the same way as before, but with pre-incubation for 3, 7, and 14 days. Next, an agar puck with mycelia was placed at the center of the plates and incubated for an additional 7 days. After that, fungal growth was classified as “-” (no growth), “+” (growth up to 7 mm), “++” (growth between 7 and 15 mm), or “+++” (growth above 15 mm) (Table 2). On the plates pre-incubated for 3 days, all fungi were able to grow, with *T. citrinoviridae* Cin-9 showing the fastest growth. However, growth was diminished compared to plates with simultaneous co-cultures. On the plates pre-incubated for 7 days, *N. oryzae* roseF7 was not able to grow, and the growth of the other fungi was reduced. Finally, on the plates pre-incubated for 14 days, none of the fungi used in the test were able to grow (Table 2). This experiment confirmed the antifungal activity of *Streptomyces* sp. S-2 against a variety of fungi. Further, it indicated that one or more products secreted by bacteria in the late phase of growth were responsible for the antifungal activity observed.

### 2.4. Genome Sequencing and Phylogeny of Streptomyces sp. S-2

**Figure 4:**
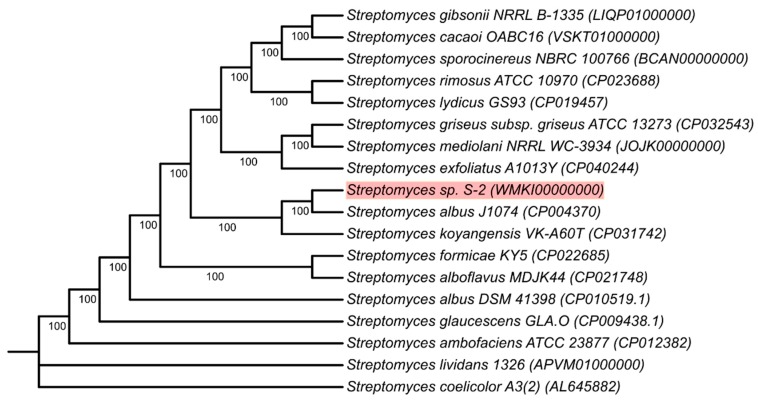
Maximum-likelihood phylogenetic tree of the *Streptomyces* sp. S-2 strain. The tree shows the phylogenetic relationships between the S-2 strain and closely related *Streptomyces* strains. The phylogenetic tree was inferred using IQ-TREE Galaxy software from an alignment of the core proteomes of 19 *Streptomyces* strains. The numbers at the nodes represent bootstrap values (% of 1000 repeats). The position of *Streptomyces* sp. S-2 strain has been highlighted in red for better visibility.

Whole-genome sequencing of *Streptomyces* sp. S-2 was performed using the Illumina MiSeq platform. The assembled sequence was deposited in GenBank under accession no. WMKI00000000. The total assembled genome of strain S-2 was 6.97 Mbp with a GC content of 73.1% and 6680 CDS. Statistical data for the genome sequence are presented in Table 3. A maximum-likelihood phylogenetic analysis was performed using the core proteomes of 18 *Streptomyces* strains. It showed the closest relation of the S-2 strain to be *S. albus* J1074 (Figure 4). Therefore, we suggest that the isolate is a new strain of the *Streptomyces* genus and is closely related to *S. albus*.

### 2.5. Prediction of Secondary Metabolites Biosynthetic Gene Clusters (BGCs) in the Streptomyces sp. S-2 Genome

The *Streptomyces* sp. S-2 genome was subjected to a prediction of secondary metabolites biosynthetic gene clusters using the anti-SMASH database. The prediction revealed that the genome encompasses a total number of 55 BGCs. Moreover, 32 of the predicted BGCs show no similarity to any of the clusters in the database (as of December 2019). Multiple clusters encode non-ribosomal peptide synthetases (NRPS) (21 clusters) and NRPS-like clusters (3); type I polyketide synthases (T1PKS) (11 clusters); type II polyketide synthases (T2PKS) (1 cluster); type III polyketide synthases (T3PKS) (1 cluster); bacteriocins (2 clusters); lanthipeptides (1 cluster); and clusters with a hybrid character (3). All of these cluster types are known to encode genes responsible for the production of compounds with biological activity, including antibiotics. Among these groups, the BGCs with no similarity to known biosynthetic clusters included 17 NRPS and NRPS-like clusters, 8 T1PKS clusters, 2 bacteriocins clusters, and 1 lanthipeptide cluster. This means that there is a heightened chance that *Streptomyces* sp. S-2 is able to produce novel antibiotics. Among the other types of BGCs with no similarity to known products were the 2 siderophores and 2 terpenes. A detailed analysis of the anti-SMASH results, which are reported in Table 4, showed the presence of BGCs typical for the *Streptomyces* genus: those used for the synthesis of geosmin, a compound responsible for the earthy odor, and desferrioxamine B, a siderophore involved in iron chelation. A BGC similar to that used for the synthesis of osmolyte ectoine (75% similarity) is present, which is not surprising given the arid environment of the chimney’s black soot. There is also a number of BGCs with moderate-to-low similarity to those that encode antibiotics, fungicides, and other bioactive substances (Table 4).

**Figure 5:**
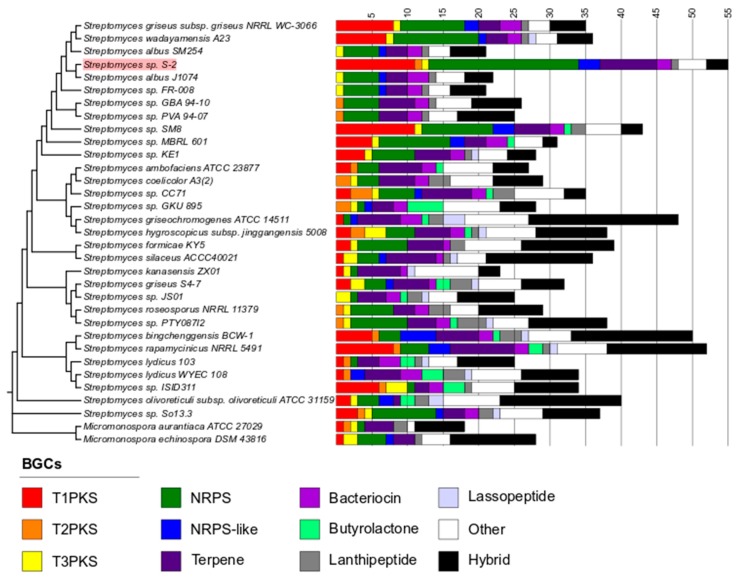
Comparison of the number and types of BGCs found in the genomes of closely related strains and strains with a high number of BGCs reported in the literature. The BGCs in each genome were predicted using the anti-SMASH database. The “Hybrid” type clusters that were predicted to belong to more than one type of BGC were pooled. The BGCs that were predicted to belong to the siderophore, ectoine, thiopeptide, indole, melanin, or linardin types which were pooled in “Other”. The position of *Streptomyces* sp. S-2 has been highlighted in red for better visibility.

The number of BGCs from *Streptomyces* sp. S-2 was compared to the number of BGCs from other members of the genus (Figure 5 and Table S2). The comparison showed that *Streptomyces* sp. S-2 excels in terms of the number of BGCs. What is worth noticing, is that the genome of strain S-2 is enriched in NRPS and T1PKS clusters in comparison to other *Streptomyces*, especially phylogenetically close companions. At the same time, only three clusters have low similarity to the hopene synthesis pathway, the compound that might play a role in adaptation to environments with high temperatures. Interestingly, the number of BGCs in the S-2 genome exceeds more than twice the number of BGCs identified in its close relatives, *S. albus* J1074 (22) and *S. albus* SM254 (21) (Figure 5). This number is also higher by more than 20 than the number of BGCs in *S. wadayamensis* A23 (36), another closely related *Streptomyces* strain.

## 3. Discussion

We isolated a new *Streptomyces* sp. S-2 strain from black soot after the combustion of hard coal. The black soot sample was collected from an in-use household chimney. To our knowledge, this is the first case of a microorganism being isolated from black soot. Phenotype fingerprinting using a GEN III microplate assay revealed that the S-2 strain can use a variety of compounds as a source of carbon and energy. This is not surprising, as high metabolic potential is a characteristic feature of the *Streptomyces* genus [2]. The assay also showed that S-2 is moderately halotolerant (with the ability to grow at 8% NaCl concentration) and can grow in the presence of desiccants. This suggests the strain has the ability to be viable in environments with low water potential, such as in chimney soot. A number of other halophilic or halotolerant *Streptomyces* strains have been previously isolated, and clearly this feature depends on environmental conditions [26,56,57]. The S-2 strain shows strong antifungal activity against plant pathogens and filamentous fungi potentially dangerous to humans. We observed the inhibition of fungal growth by S-2 early in the simultaneous co-culture with tested fungi, although with different levels for different fungal genera. Notably, *Streptomyces* produce antibiotics mainly in the later phase of growth, when the sporulation process takes place [58,59]. Moreover, both *T. citrinoviridae* Cin-9 and *A. niger* may respond to S-2 secondary metabolites through the secretion of enzymes which counteract the inhibitory effect of compounds found in the environment. Members of the *Trichoderma* genus are also used as biocontrol agents, and as such have antimicrobial activity and are resistant to a plethora of adverse compounds [60,61,62]. In our experiment, both bacteria and fungi were inoculated simultaneously. This could have potentially influenced the degree of the observed inhibitory effect, especially in the case of fast-growing fungi (i.e. members of the *Trichoderma*, *Aspergillus*, or *Nigrospora* genera). Nevertheless, significant inhibition on day 2 of the co-culture indicated that *Streptomyces* sp. S-2 has strong antifungal properties. In co-cultures with pre-cultured S-2, the effect was even more pronounced, resulting in the complete inhibition of fungal growth on plates with bacteria pre-cultured for 7 and 14 days for *N. oryzae* roseF7 and for all tested fungi, respectively. Recently, there have been a number of reports of new *Streptomyces* strains isolated from extreme and non-conventional environments that are showing antimicrobial activity [1,13,24,25]. *Streptomyces* strains have also been previously studied for their potential to protect plants against fungal infections [2,35]. One example is *S. lydicus* WYEC 108, a bioactive compound in antifungal product sold in the United States, for agricultural use [49]. Another example is *S. violaceusniger* YCED9, which produces antifungal agents able to combat various plant pathogens, including members of *Fusarium*, *Phythium*, and *Phytophthora* genera [50]. On the other hand, field trials to use *Streptomyces* strains as a biocontrol agent in banana wilt disease are have not yet been reported [35].

Sequencing has shown that the size of the S-2 genome (<7 Mbp) as well as number of coding sequences (CDS) (<7000) are on the lower end for *Streptomyces* genus whose members’ genomes are 8.6 Mbp on average and contain more than 7000 CDS [5,13]. At the same time, the genome mining of S-2 revealed 55 BGCs. A higher-than-average number of BGCs (20-30) that has been reported for mesophilic *Streptomyces* [13,63]. It is also more BGCs than identified in *Streptomyces* isolated from extreme environments, like Artic soil, caves or coal mine soil [1,13,24]. Interestingly, the number of BGCs in the S-2 genome exceeds those identified in genomes of closely related strains. Recent work by Chevrette et al. [25] has shown that the ecology of a microorganism influences its metabolic potential. This could be the explanation for the high number of BGCs in the S-2 genome, giving the strain an advantage in the competition for scarce nutrients in a black soot environment. Similarly, two strains of *Streptomyces*, sp. ICC1 and ICC4, isolated from the Iron Curtains Cave have been reported to have a higher number of BGCs than their closest terrestrial counterpart, *S. lavendulae*, has [24]. To the best of our knowledge, only two strains with a higher number of BGCs than S-2 have been described: *S. griseochromogenes* ATCC-14511 [13] and *Streptomyces* sp. GMR22 [64], with 59 and 63 BGCs respectively. However, those analyses were performed with an older version of the anti-SMASH database. A re-analysis with anti-SMASH version 5.1.0 revealed the presence of a lower number of BGCs in *S. griseochromogenes* ATCC-14511 than before, 47 versus 59. Due to not being able to access the genome of *Streptomyces* sp. GMR22, we were unable to assess the number of BGCs in this strain. Moreover, in our study, 32 out of 55 identified BGCs were classified as either NRPS (21 clusters) or T1PKS (11 clusters). Both groups of BGCs are known to encode proteins involved in biosynthesis and the processing of secondary metabolites with various antibiotic activities [65]. In other microorganisms, NRPS are responsible for synthesis of compounds such as actinomycin, vancomycin, and coelibactin [66,67,68], and T1PKS produce compounds such as rapamycin, cyphomycin, and glutaramides [18,25,69]. On the other hand, phenotype fingerprinting using a microplate assay showed that the S-2 strain was susceptible to several antibacterial antibiotics targeting protein synthesis. *Streptomyces* sp. myrophorea McG1, which has antibacterial activity, has been shown to be resistant to a number of antibiotics, including those targeting protein synthesis [27]. This might suggest a lack of antibacterial activity in *Streptomyces* sp. S-2. However, in that study, the resistance to some antibiotics of the McG1 strain was established after 6 days of culture, and the test was performed using different methodology [27]. The high number of BGCs, as well as the prevalence of NRPS and T1PKS clusters, distinguish the S-2 strain from other *Streptomyces* strains, especially considering its relatively small genome size. Finally, 32 of the identified BGCs show no similarity to any clusters present in the database, and 10 show only low similarity (below 20%). This seems particularly interesting, as it may lead to the production of novel compounds. The same has been suggested for BGCs with low overall similarities to known compounds in other *Streptomyces* [24].

The results reported in this study point to the capability of *Streptomyces* sp. S-2 to produce a large number of secondary metabolites. The high numbers of clusters in the NRPS and T1PKS groups indicate that many of its produced metabolites may have antibiotic activity. A number of them might be novel compounds and may be beneficial in medicine and industry. Secondary metabolites produced by *Streptomyces* sp. S-2, aside from antibiotic activity, might display antitumor, anti-inflammatory, or antioxidant activities, or function as insecticides. Such activities have been previously reported in the *Streptomyces* genus [2,10,70,71,72]. Considering the biosynthetic potential of the S-2 strain, future research focusing on investigation of these activities is ensured.

It should be noted that some biosynthetic clusters might be dormant and might only be expressed under specific conditions or upon activation by external factors. In members of *Streptomyces*, it has been shown that a co-culture with other microorganisms might stimulate the production of antibiotics [22]. A two-component signaling cascade is known to be present in these bacteria and might induce the expression of secondary metabolites under specific environmental conditions, e.g. nutrient deprivation [21]. Additionally, the activation of dormant BGCs through genetic manipulation resulting in the production of a new antimicrobial compound has recently been reported [23]. The results from co-cultures with pre-cultured S-2 suggest a constitutive production of antifungal agents by the strain. Nevertheless, a detailed examination of BGCs expression and the activities of the compounds produced by S-2 should be the focus of future research, as this could elucidate the full biotechnological potential of the strain.

## 4. Materials and Methods 

### 4.1. Microbial Culture Media

Minimal salt medium (MSM) was prepared according to Malik et al. [73] to isolate the microorganisms from the black soot and cinder samples. For media supplementation, hard coal dust was obtained by grinding hard coal pieces using a ball mill. For media solidification ultra-pure agar was added for a final concentration of 1.5% (Select Agar, ThermoFisher Scientific, Waltham, MA, USA). For the fungal cultures potato dextrose agar (PDA) medium was used. For antifungal activity, nutrient agar (NA) and Mueller—Hinton agar (MHA) media were used. For the selection of transformants Luria–Bertani (LB) medium supplemented with 1.5% agar, 50 μg/mL ampicillin (Sigma-Aldrich, Darmstadt, Germany), 40 μg/mL X-gal and 0.1 mM isopropyl β-D-1-thiogalactopyranoside (IPTG) was used (both from A&A Biotechnology, Gdynia, Poland). For liquid cultures, nutrient broth (NB) and LB media were used for S-2 and transformed *E. coli* DH5α, respectively. All media were purchased from BTL sp. z o.o., Łódź, Poland, unless stated otherwise.

### 4.2. Sampling and Strains Isolation

The black soot sample was collected from a household chimney under active use (50.191846 N, 19.108628 E) on 19 December 2017 in the city of Mysłowice, Poland. At the time of collection, the chimney interior had a temperature of 30 °C and was exhausting smoke from hard coal combustion. The soot sample was scraped off the chimney wall with a sterilized spoon into a sterile 50 mL tube and transferred to the laboratory. A sample of cinder after hard coal combustion was collected from under the household’s hearth on the same day and in the same location as the black soot sample. The top layer of cinder was scraped off the pile, and the exposed cinder was collected into a sterile 50 mL tube with a sterilized spoon and transferred to the laboratory. 1 g of each sample was spread onto an MSM agar plate supplemented with 1% (w/v) hard coal dust. Plates were incubated at 30 °C. After 2 weeks, the plates were investigated, and bacterial and fungal strains were passed onto MSM agar plates supplemented with 2% (MSM-C2) hard coal dust, in order to isolate pure cultures. Strains S-2 from the black-soot-inoculated plate and Cin-9 from the cinder-inoculated-plate were chosen for subsequent experiments. S-2 resembled the morphology of Actinobacteria, and Cin-9 resembled the morphology of *Trichoderma*.

The microscopic morphology of isolated bacteria was investigated by using Gram staining. A colony was suspended in a drop of 0.85% NaCl and smeared on a microscope slide, air-dried, and heat fixed. Then, the specimen was stained through flooding, as follows: crystal violet for 3 min, Gram’s iodine for 2 min, 20% ethanol for 30 s, and carbol fuchsin for 30 s, followed by air-drying. A microscopic observation of the specimen was performed using a PrimoStar microscope (Carl Zeiss, Jena, Germany) with a Primo Plan-ACHROMAT 100x NA1.25 objective (Carl Zeiss, Jena, Germany) under immersion. Pictures were recorded using an AxioCam ERc 5s (Carl Zeiss, Jena, Germany) and processed using Fiji software, (version 1.52p) [74]

On 18 August 2018, garden rose leaves showing signs of fungal infection were collected into a sterile 50 mL tube and transferred to the laboratory to isolate fungi. The leaves were surface sterilized in a microbiological flow cabinet by washing them in 70% ethanol, followed by washing them in 5% commercial bleach solution (ACE, Procter & Gamble, Cincinnati, OH, USA) and then washing them twice in deionized water. Next, the leaves were placed on PDA plates and incubated at 22 °C. After 7 days of incubation, the colonies growing from the leaves were placed on fresh PDA plates and incubated at 22 °C in order to isolate the pure cultures. The strain isolate *N. oryzae* roseF7 was used for subsequent experiments. 

Juniper branches with “slime mold” were collected into a sterile 50 mL tube and transferred to the laboratory. Pieces of branches with “slime mold” were placed on PDA plates in a microbiological flow cabinet and incubated at 22 °C. After 7 days of incubation, the colonies growing from the inoculated material were placed onto fresh PDA plates incubated at 22 °C in order to isolate the pure cultures. The *C. coatesieae* junF9 strain was used for subsequent experiments.

### 4.3. Microorganism Strains

The *Aspergillus niger* used in this study was obtained from the Institute of Biology, Biotechnology and Environmental Protection, University of Silesia in Katowice. The *Fusarium avenaceum* strain isolated from the environmental sample was obtained from the Institute of Biotechnology for Agriculture-Food Industry, Poland. For the transformation with ITS, amplicon *Escherichia coli* DH5α strain was used.

### 4.4. DNA Isolation and Sequencing

After 8 days of culture on the MSM-C2 agar plate, the S-2 strain was loop inoculated in 5 mL of NB medium and incubated at 30 °C, with shaking at 120 rpm for 72 h. Next, the cells were pelleted through centrifugation at 10,000 rcf and used for DNA extraction. Genomic DNA was extracted using a PowerSoil DNeasy Kit (MoBio, Carlsbad, CA, USA) according to the manufacturer’s protocol, with the exception that instead of soil, cell pellet was used as the sample. The integrity of the DNA was examined by agarose gel electrophoresis. The whole genome sequencing was performed by MicrobesNG (Birmingham, United Kingdom) using an Illumina MiSeq platform with 2 × 250-bp paired-end reads. The results of the sequencing were put through a standard MicrobesNG analysis pipeline.

Isolated fungal strains were cultured on PDA plates at 22 °C for 8 days, except for the Cin-9 strain, which was cultured at 30 °C. Next, the mycelia were harvested and homogenized using a sterile ceramic mortar and pestle. The homogenates were used for DNA extraction, which was performed with a DNeasy PowerLyzer PowerSoil Kit (MoBio, Carlsbad, CA, USA) according to the manufacturer’s protocol. For fungal strain identification, the ITS region was amplified using an ITS1F primer (5’-TCCGTAGGTGAACCTGCGG-3’) and ITS4R primer (5’-TCCTCCGCTTATTGATATGC-3’). In each sample, the reaction mix was composed of DreamTaq polymerase, 0.25 μL (1.25 U), 10x DreamTaq buffer, 5 μL, dNTPs mix, 5 μL (2 mM of each dNTP), primers, 0.25 μL of each (0.5 μM each), template, and nuclease-free water to fill to 50 μL. All reagents were purchased from Thermo Fisher, Waltham, MA, USA, except for the primers, which were ordered from Sigma–Aldrich, Darmstadt, Germany. Between 10 pg and 1 μg of fungal DNA was used as a template. PCR was performed as follows: initial denaturation at 95 °C for 10 min, followed by 35 cycles of denaturation at 95 °C for 30 s, primers annealing at 57 °C for 30 s, elongation at 72 °C for 1 min, and final elongation at 72 °C for 15 min. PCR products were purified using a Clean Up Ax Kit (A&A Biotechnology, Gdynia, Poland). Purified products were ligated into a pJET1.2 vector using a Clone JET PCR Cloning Kit (Thermo Fisher, Waltham, MA, USA) and subsequently transformed into chemically competent *E. coli* DH5α. Positive transformants were selected using a blue-white screen on LB plates supplemented with ampicillin (50 μg/mL), X-gal (40 μg/mL), and IPTG (0.1 mM). Vectors from positive clones were isolated using a GeneJet Plasmid MiniPrep Kit (Thermo Fisher Scientific, Waltham, MA, USA) according to the manufacturer’s protocol. Insert sequencing was performed by Macrogen (Amsterdam, Netherlands) using the Sanger method with a pJET1.2 forward primer (5′-CGACTCACTATAGGGAGAGCGGC-3′) and a pJET1.2 reverse primer (5′-AAGAACATCGATTTTCCATGGCAG-3′). Fungal strains were identified using by retrieving sequences of ITS1, 5.8S rRNA, ITS2, and partial large subunit rRNA fragment of closely related strains using a Blast tool [75].

### 4.5. Phylogenetic Analysis of Streptomyces sp. S-2

The phylogenetic tree was created by extracting and aligning the core proteomes of the S-2 and 18 *Streptomyces* strains (list of strains available in Table S3) using M1CR0B1AL1Z3R [76]. Poorly aligned regions were removed using Gblocks (version 0.91b) [77], which yielded 176,196 amino acids for 18 genomes. A maximum-likelihood phylogenetic tree was inferred using IQ-TREE Galaxy (version 1.5.5.3) with default settings and 1000 bootstraps [78]. Finally, the phylogenetic tree was visualized using the iTOL Interactive tree of life tool (version 5.5) [79], and is available at https://itol.embl.de/tree/19348172168181091583040528.

### 4.6. Secondary Metabolite Gene Clusters Prediction

The prediction of biosynthetic gene clusters for secondary metabolite synthesis was performed using Antibiotic and Secondary Metabolite Analysis Shell (anti-SMASH) version 5.1.0 [80,81]. For the characteristic of the cluster types predicted to be present in each genome, clusters assigned to more than one group were pooled in the hybrid group. The strains used in the anti-SMASH analysis were close relatives of S-2, based on a Blast search, or were strains with high a number of BGCs, as reported in literature. A tree of 32 high-quality bacterial genomes (Table S4) was generated by retrieving 31 single-copy, housekeeping genes from each genome using AmphoraNet [82]. The sequences of each individual marker gene were aligned in Geneious Prime software (version 2019.1.1, Geneious, Auckland, New Zealand) with default parameters and the alignments were concatenated. Poorly aligned positions were removed utilizing Gblocks software (version 0.91b). A maximum-likelihood phylogenetic tree was inferred using MEGA 7 software [83]. To support the phylogenetic tree, a bootstrap test with 1000 replicates was performed. Finally, the phylogenetic tree was visualized using the iTOL Interactive tree of life tool and is available at https://itol.embl.de/tree/1551582516147091575216186#.

### 4.7. Phenotype Fingerprinting

For phenotype fingerprinting, *Streptomyces* sp. S-2 was cultured on an MSM-C2 agar plate for 8 days. Next, cells were scraped and suspended in IFIB solution (BiOLOG, Hayward, CA, USA) until a transmittance of 98% was achieved. The solution was mixed, and a GENIII plate (BiOLOG, Hayward, CA, USA) was inoculated with 100 μL of cell suspension per well. The absorbance was measured at λ = 590 nm for each well, and the plate was incubated at 30 °C for 48 h. After incubation, the absorbance of each well was measured again. The readings were corrected for the absorbance of the negative control by subtracting the value of the A1 well from the rest of the wells. For negative results, the value was assumed to be zero. Subsequently, readings after 48 h were corrected for the initial absorbance measured after inoculation. Wells with negative values were assumed to be zero. The experiment was repeated three times, the values for each well were averaged, and the standard deviation was calculated.

### 4.8. Antifungal Activity Tests

To test antifungal activity, *Streptomyces* sp. S-2 cultured for 8 days on an MSM-C2 agar plate was spot-inoculated at three equally distributed spots, 20 mm from the test plate center. Next, an 8 mm agar puck with mycelia was drilled out of the PDA plates with fungi cultured for 7 days at 22 °C and placed in the center of the test plate (Figure S1). For controls, plates were inoculated in the same fashion, but were prepared with either S-2 or fungi. For *A. niger*, *F. avenceum*, *N. oryzae* roseF7, and *C. coatesieae* junF9, MHA plates were used. For *T. citrinoviridae* Cin-9, instead of MHA, NA plates were used, as the growth of this fungi is suppressed on MHA plates. Three control plates for each fungus and the S-2 were prepared, and at least 15 co-culture plates for each pair were prepared. Plates were incubated at 25 °C, except for *T. citrinoviridae* Cin-9, which was incubated at 30 °C. The plates were investigated every day for 14 days, and pictures were taken. Antifungal activity was analyzed by measuring mycelium growth towards the plate border between colonies and towards the S-2 colonies (Figure S1). Growth distances were measured using Fiji software [74]. A one-way ANOVA test was carried out to determine the significance of differences in the growth distances, followed by Tukey’s post hoc test (*p* ≤ 0.05). Statistical analyses were conducted using Statistica version 13 (Statsoft, Kraków, Poland). For the second antifungal activity experiment, the plates were prepared in a similar way, with the exception that inoculated S-2 was precultured for 3, 7, and 14 days before the placement of an agar puck with mycelia in the center of the plate. The plates were then incubated for 7 days and classified based on the growth of the mycelia. For each co-culture, the experiment was repeated three times.

## 5. Conclusions

We have isolated the *Streptomyces* sp. S-2 strain from black soot after hard coal combustion collected from an in-use household chimney. This is the first report of microorganism isolation from black soot. The isolated strain features strong antifungal activity and inhibits the growth of plant pathogens and filamentous fungi potentially dangerous to humans. It will be interesting to see if the strain can protect plants from fungal infections, a study that is currently ongoing. Genome sequencing and anti-SMASH analysis revealed that although this is not a new species, it contains a very high number of BGCs present in its genome, a feature that distinguishes it from closely related species. This indicates the high metabolic potential of S-2 and might increase the strain fitness in harsh environments e.g. black soot. Many of the detected BGCs show no, or low (below 20%) similarity to the clusters present in the database, increasing the possibility that S-2 might be a source of novel compounds for biomedicine and industry. Future, detailed studies on the biosynthetic abilities of *Streptomyces* sp. S-2 will elucidate its full biotechnological potential. This work shows that studying previously neglected and extreme environments might be a source of new strains and compounds, with a variety of biotechnological implications.

## Figures and Tables

**Table 1:** *Streptomyces* sp. S-2 phenotypic fingerprint based on a GEN III microplate assay after 48 h of incubation.

**Carbon Source Assay**							
**Compound**	**OD_590_**	**Compound**	**OD_590_**	**Compound**	**OD_590_**	**Compound**	**OD_590_**
**Saccharides**							
α-D-Glucose	0.214 ± 0.064	D-Galactose	0.089 ± 0.046	D-Mannose	0.120 ± 0.063	D-Fructose	0.196 ± 0.047
D-Cellobiose	0.145 ± 0.037	Gentiobiose	0.320 ± 0.151	D-Turanose	0.036 ± 0.026	N-Acetylo-D-Glucosamine	0.082 ± 0.44
Dextrin	0.155 ± 0.088						
**Saccharide derivatives**							
N-Acetyl-β-D-Mannosamine	0.077 ± 0.023	D-Glucose-6-Phosphate	0.086 ± 0.033	D-Fructose-6-Phosphate	0.063 ± 0.053	N-Acetyl- Neuraminic Acid	0.183 ± 0.021
Mucic Acid	0.065 ± 0.041						
**Glycosides and polyols**							
β-D-Methyl-D-Glucoside	0.098 ± 0.049	D-Salicin	0.175 ± 0.030	Glycerol	0.112 ± 0.048	D-Mannitol	0.067 ± 0.049
**Organic Acids**							
Formic Acid	0.291 ± 0.082	Acetic Acid	0.249 ± 0.029	Propionic Acid	0.237 ± 0.021	Citric Acid	0.104 ± 0.076
L-Lactic Acid	0.315 ± 0.224	D-Gluconic Acid	0.595 ± 0.127	D-Malic Acid	0.133 ± 0.024	L-Malic Acid	0.420 ± 0.083
Acetoacetic Acid	0.114 ± 0.031	Bromo-Succinic Acid	0.193 ± 0.084	Methyl Pyruvate	0.218 ± 0.021	α-Hydroxy-Butiric Acid	0.318 ± 0.082
β-Hydroxy-D,L-Butyric Acid	0.267 ± 0.024	α-Keto-Butyric Acid	0.248 ± 0.042	γ-Amino-Butyric Acid	0.102 ± 0.003	D-Lactic Acid Methyl Ester	0.176 ± 0.041
**Amino Acids**							
L-Alanine	0.115 ± 0.087	L-Histidine	0.199 ± 0.047	L-Aspartic Acid	0.134 ± 0.098	L-Glutamic Acid	0.125 ± 0.069
Glycyl-L-Proline	0.095 ± 0.020						
**Others**							
Gelatin	0.220 ± 0.009	Inosine	0.102 ± 0.026	Tween 40	0.266 ± 0.102		
**CHEMICAL SENSITIVITY ASSAY**							
pH 6	0.487 ± 0.107	1% NaCl	1.018 ± 0.294	4% NaCl	0.636 ± 0.151	8% NaCl	0.349 ± 0.103
1% Sodium Lactate	0.508 ± 0.146	Guanidine HCl	0.364 ± 0.039	Nalidixic Acid	0.456 ± 0.107	Aztreonam	0.250 ± 0.085
Sodium Butyrate	0.467 ± 0.160						

The values represent bacterial growth measured as optical density (OD) at a 590 nm wavelength. The values are the mean and standard deviation error calculated from three individual biological repeats.

**Table 2:** Inhibition of fungal growth on plates with pre-cultured *Streptomyces* sp. S-2.

Fungus species	pre-cultured *Streptomyces* sp. S-2		
	3 days	7 days	14 days
*Fusarium avenaceum*	++	+	-
*Aspergillus niger*	++	+	-
*Trichoderma citrinoviridae* Cin-9	+++	++	-
*Nigrospora oryzae* RoseF7	++	-	-
*Curvularia coatesieae* JunF9	++	+	-

An agar puck with mycelia was placed in the middle of plates with three colonies of Streptomyces sp. S-2. The plates were incubated for 7 days, and the growth of the fungi was measured as the radius between the puck and center of the S-2 colony: “-” (no growth), “+” (growth up to 7 mm), “++” (growth between 7 and 15 mm), and “+++” (growth above 15 mm).

**Table 3:** **Next Generation Sequencing** (NGS) statistical data based on a draft genome of S-2.

*Streptomyces* sp. S-2 Draft Genome	
**Number of contigs**	1799
Contigs sum (bp)	6.971375
N50	6745
Longest contig (bp)	62.211
Shortest contig (bp)	407
Average length (bp)	3875
% GC content	73.1
Number of CDS	6680

**Table 4:** The distribution of BGCs from *Streptomyces* sp. S-2.

Gene Type	Span [nt]		Most Similar Biosynthetic Gene Cluster
	From	To	
**Bacteriocin**	1	8813	-
Bacteriocin	1	1875	-
Ectoine	1	4689	Ectoine (75%)
Lanthipeptide	1	6359	-
NRPS	1	14,680	-
NRPS	1	7664	-
NRPS	1	7000	-
NRPS	1	5847	-
NRPS	1	3863	-
NRPS	1	3784	-
NRPS	1	3409	-
NRPS	1	3289	-
NRPS	1	3179	-
NRPS	1	3061	-
NRPS	1	2796	-
NRPS	1	2701	-
NRPS	1	2511	-
NRPS	1	1858	-
NRPS	8528	41,043	Diisonitrile entibiotic SF2768 (66%)
NRPS	1	20,858	Mannopetimycin (37%)
NRPS	1	27,653	Surugamide A / Surugamide D (19%)
NRPS	1	6554	Curacomycin (18%)
NRPS	1	30,250	Dechlorocuracomycin (16%)
NRPS	1	7538	Desotamide (9%)
NRPS	1	10,029	Mannopeptimycin (7%)
NRPS-like	1	7313	-
NRPS-like	1	4785	-
NRPS-like	1	1417	-
Hybrid (NRPS-like, T1PKS)	1	22,016	Levorin A3/C06690/FR-008-III/candicidin A/UNII-AP5PEF5W7U (66%)
siderophore	4004	10,744	-
siderophore	1	1673	-
siderophore	1	10,733	Desferrioxamine B (100%)
T1PKS	1	18,159	-
T1PKS	1	6047	-
T1PKS	1	6025	-
T1PKS	1	5494	-
T1PKS	1	4600	-
T1PKS	1	3778	-
T1PKS	1	1980	-
T1PKS	1	1646	-
T1PKS	1	15,272	ECO-02301 (32%)
T1PKS	1	14,101	Candicidin (23%)
T1PKS	1	16,488	Reedsmycins (20%)
Hybrid (T1PKS, NRPS)	1	13,542	Antimycin (20%)
Hybrid (T1PKS, NRPS)	1	13,498	Antimycin (13%)
T2PKS	1	16,805	Fredericamycin A (45%)
T3PKS	1	8472	Herboxidiene (7%)
Terpene	1	4631	-
Terpene	1	4141	-
Terpene	1	2347	Geosmin (100%)
Terpene	1	9567	Hopene (53%)
Terpene	1	6983	Isorenieratene (25%)
Terpene	1	1604	Hopene (15%)
Terpene	1	1104	Hopene (15%)
Terpene	3697	13,999	Phoshonoglycans (6%)

Biosynthetic gene clusters (BGCs) predicted based on the anti-SMASH database (% indicates the proportion of genes with similarities). TIPKS: type I polyketide synthase; TIIPKS: type II polyketide synthase; TIIIPKS: type III polyketide synthase; NRPS: non-ribosomal peptide synthase.

## Supplementary Materials

Supplementary materials can be found at https://www.mdpi.com/1422-0067/21/7/2558/s1. **Figure S1**: Schematic representation of the co-culture plate inoculation used for examination of the antifungal activity of the S-2 strain. The S-2 strain was inoculated at three equally separated points 20 mm from the center of the plate (green spots), and an agar puck with fungal mycelium was placed at the center of the plate (red spot). The double arrow lines indicate the measured distance of mycelial growth toward the plate wall (violet line) and toward the S-2 colony (blue line). For each plate, three sets of measurements were taken. **Table S1:** Complete *Streptomyces* sp. S-2 phenotypic fingerprint based on GEN III microplate assay after 48 h of incubation. **Table S2:** Anti-SMASH results for all of the strains used for the BGCs analysis. **Table S3:** List of strains used in the phylogenetic analysis, with basic statistics for their genomes and description of isolation sources. **Table S4:** List of strains used for the phylogenetic analysis in anti-SMASH analysis with basic statistics for their genomes and description of isolation sources.

## Abbreviations

BGCs	Biosynthetic gene clusters
NRPS	Non-ribosomal peptide synthase
T1PKS	Type 1 polyketide synthase
