# Supplementary material for: Genome Mining Revealed a High Biosynthetic Potential for Antifungal Streptomyces sp. S-2 Isolated from Black Soot
Source: Int J Mol Sci. 2020 Apr 7;21(7):2558. doi: 10.3390/ijms21072558 (PMC7177978; doi:10.3390/ijms21072558)
Supplement: Supplementary file 1 [file ijms-21-02558-s001.zip › S-2_Table_S1.docx]

**Table S1.** Complete *Streptomyces* sp. S-2 phenotypic fingerprint based on GEN III microplate assay after 48 h of incubation.

| **Carbon Sources Utilization Assay** | | | | | | | |
| --- | --- | --- | --- | --- | --- | --- | --- |
| **Compound** | **OD_590_** | **Compound** | **OD_590_** | **Compound** | **OD_590_** | **Compound** | **OD_590_** |
| Dextrin | 0,155  ± 0,088 | D-Maltose | 0,000  ± 0,000 | D-Trehalose | 0,000  ± 0,000 | D-Cellobiose | 0,145  ± 0,037 |
| Gentiobiose | 0,320  ± 0,151 | Sucrose | 0,000  ± 0,000 | D-Turanose | 0,036  ± 0,026 | Stachyrose | 0,000  ± 0,000 |
| D-Raffinose | 0,000  ± 0,000 | α-D-Lactose | 0,000  ± 0,000 | D-Melibiose | 0,000  ± 0,000 | β-D-Methyl-D-Glucoside | 0,098  ± 0,049 |
| D-Salicin | 0,175  ± 0,030 | N-Acetylo-D-Glucosamine | 0,082  ± 0,44 | N-Acetyl-β-D-Mannosamine | 0,077  ± 0,023 | N-Acetyl-D-Galactosamine | 0,0000  ± 0,0000 |
| N-Acetyl- Neuraminic Acid | 0,183  ± 0,021 | α-D-Glucose | 0.214  ± 0,064 | D-Mannose | 0,120  ± 0,063 | D-Fructose | 0,196  ± 0,047 |
| D-Galactose | 0,089  ± 0,046 | 3-Methyl Glucose | 0,000  ± 0,000 | D-Fucose | 0,000  ± 0,000 | L-Fucose | 0,000  ± 0,000 |
| L-Rhamnose | 0,000  ± 0,000 | Inosine | 0,102  ± 0,026 | D-Sorbitol | 0,000  ± 0,000 | D-Mannitol | 0,067  ± 0,049 |
| D-Arabitol | 0,000  ± 0,000 | Myo-Inositol | 0,000  ± 0,000 | Glycerol | 0,112  ± 0,048 | D-Glucose-6-Phosphate | 0,086  ± 0,033 |
| D-Fructose-6-Phosphate | 0,063  ± 0,053 | D-Aspartic Acid | 0,000  ± 0,000 | D-Serine | 0,000  ± 0,000 | Gelatin | 0,220  ± 0,009 |
| Glycyl-L-Proline | 0,095  ± 0,020 | L-Alanine | 0,115  ± 0,087 | L-Arginine | 0,000  ± 0,000 | L-Aspartic Acid | 0,134  ± 0,098 |
| L-Glutamic Acid | 0,125  ± 0,069 | L-Histidine | 0,199  ± 0,047 | L-Pyroglutamic Acid | 0,000  ± 0,000 | L-Serine | 0,000  ± 0,000 |
| Pectin | 0,000  ± 0,000 | D-Galacturonic Acid | 0,000  ± 0,000 | L-Galactonic Acid Lactone | 0,000  ± 0,000 | D-Gluconic Acid | 0,595  ± 0,127 |
| D-Glucuronic Acid | 0,000  ± 0,000 | Glucuronamide | 0,000  ± 0,000 | Mucic Acid | 0,065  ± 0,041 | Quinic Acid | 0,000  ± 0,000 |
| D-Saccharic Acid | 0,000  ± 0,000 | p-Hydroxy-Phenylacetic Acid | 0,000  ± 0,000 | Methyl Puryvate | 0,218  ± 0,021 | D-Lactic Acid Methyl Ester | 0,176  ± 0,041 |
| L-Lactic Acid | 0,315  ± 0,224 | Citric Acid | 0,104  ± 0,076 | α-Keto-Glutaric Acid | 0,000  ± 0,000 | D-Malic Acid | 0,133  ± 0,024 |
| L-Malic Acid | 0,420  ± 0,083 | Bromo-Succinic Acid | 0,193  ± 0,084 | Tween 40 | 0,266  ± 0,102 | γ-Amino-Butyric Acid | 0,102  ± 0,003 |
| α-Hydroxy-Butiric Acid | 0,318  ± 0,082 | β-Hydroxy-D,L-Butyric Acid | 0,267  ± 0,024 | α-Keto-Butyric Acid | 0,248  ± 0,042 | Acetoacetic Acid | 0,114  ± 0,031 |
| Propionic Acid | 0,237  ± 0,021 | Acetic Acid | 0,249  ± 0,029 | Formic Acid | 0,291  ± 0,082 |  |  |
| **Chemical Sensitivity Assay** | | | | | | | |
| **Compound** | **OD_590_** | **Compound** | **OD_590_** | **Compound** | **OD_590_** | **Compound** | **OD_590_** |
| pH 6 | 0,487  ± 0,107 | pH 5 | 0,000  ± 0,000 | 1% NaCl | 1,018  ± 0,294 | 4% NaCl | 0,636  ± 0,151 |
| 8% NaCl | 0,349  ± 0,103 | 1% Sodium Lactate | 0,508  ± 0,146 | Fusidic Acid | 0,000  ± 0,000 | D-Serine | 0,000  ± 0,000 |
| Troleandomycin | 0,000  ± 0,000 | Rifamycin SV | 0,000  ± 0,000 | Minocycline | 0,000  ± 0,000 | Lincomycin | 0,000  ± 0,000 |
| Guanidine HCl | 0,364  ± 0,039 | Niaproof 4 | 0,000  ± 0,000 | Vancomycin | 0,000  ± 0,000 | Tetrazolium Violet | 0,000  ± 0,000 |
| Tetrazolium Blue | 0,000  ± 0,000 | Nalidixic Acid | 0,456  ± 0,107 | Lithium Chloride | 0,800  ± 0,167 | Potassium Tellurite | 0,000  ± 0,000 |
| Aztreonam | 0,250  ± 0,085 | Sodium Butyrate | 0,467  ± 0,160 | Sodium Bromate | 0,619  ± 0,212 |  |  |

The values are representation of bacterial growth measured as OD at 590 nm wavelength. The values are mean with standard deviation error calculated from 3 individual biological repeats.
