# Supplementary figures and images for: Genome Mining Revealed a High Biosynthetic Potential for Antifungal Streptomyces sp. S-2 Isolated from Black Soot
Source: Int J Mol Sci. 2020 Apr 7;21(7):2558. doi: 10.3390/ijms21072558 (PMC7177978; doi:10.3390/ijms21072558)

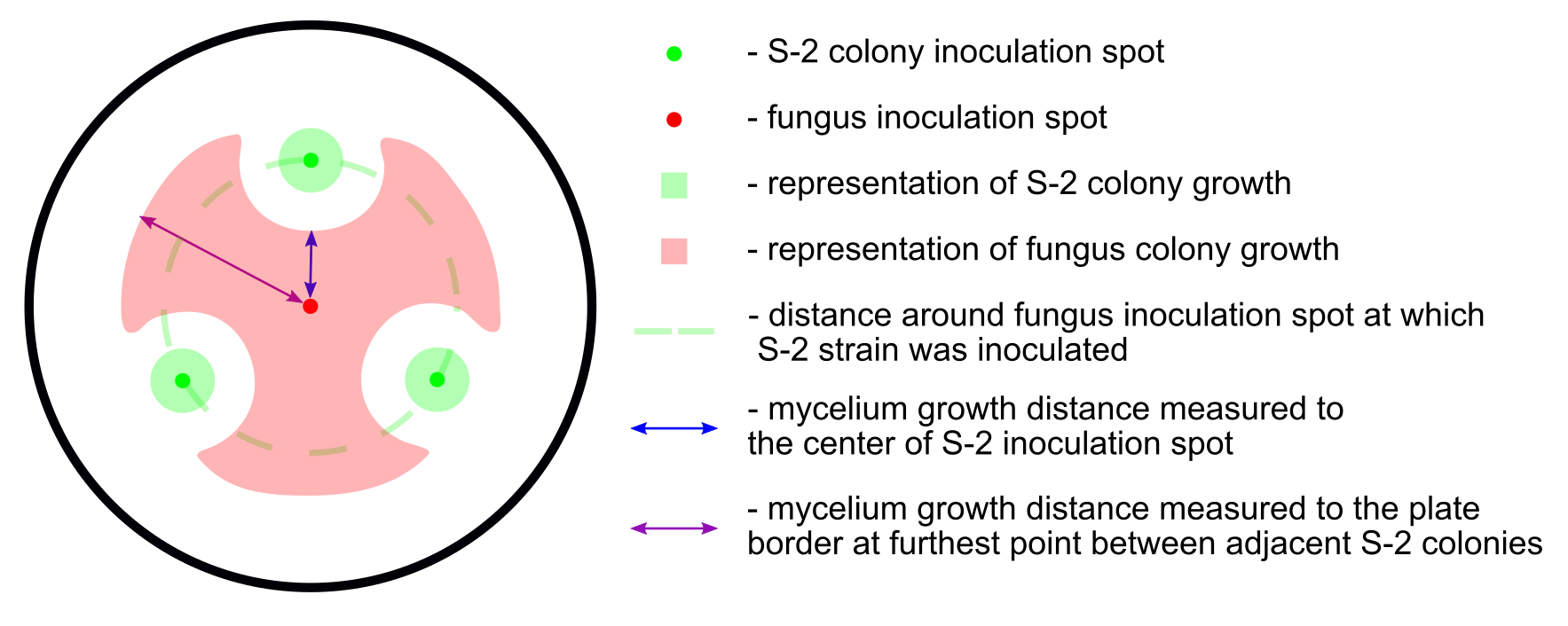

Supplement: Supplementary file 1 [file ijms-21-02558-s001.zip › FIGURE_S1.tif]
